# Questioning the association between prophylactic antibiotics and improved prognosis in gastric cancer immunotherapy

**DOI:** 10.1093/oncolo/oyag093

**Published:** 2026-03-16

**Authors:** Yusuf Ilhan, Tolga Kosecı

**Affiliations:** Antalya City Hospital, Department of Medical Oncology, Antalya, 07080, Turkey; Çukurova University, Faculty of Medicine, Department of Medical Oncology, Adana, 01330, Turkey

We read with great interest the article by Zhang et al. titled “Prophylactic antibiotic use is associated with better clinical outcomes in gastric cancer patients receiving immunotherapy”.^1^ The authors are commended for exploring the relationship between prophylactic antibiotic use and immunotherapy outcomes in metastatic gastric cancer. However, several aspects of the study warrant further discussion.

Interpretation of the reported survival benefit requires caution due to potential baseline imbalances between groups. The median overall survival (OS) was 22.6 months in patients receiving prophylactic antibiotics and 14.7 months in those who received no antibiotics (HR: 0.57; 95% CI, 0.39-0.83), while outcomes were poorest in patients treated for infection.^1^ Substantial differences in treatment stage were observed between groups: 95.7% of patients in the prophylactic antibiotic group were included during first-line therapy, whereas only 60.6% of patients in the non-antibiotic group were in first-line treatment, with 39.4% having already received second- or third-line therapy. Because treatment line strongly influences prognosis, this imbalance introduces significant heterogeneity and may confound survival comparisons. Importantly, antibiotic use in retrospective studies is not randomly assigned and may be influenced by clinical status, treatment stage, and physician decision-making, further increasing the risk of selection bias. Patients who did not receive prophylactic antibiotics and who had poorer survival had three times more liver metastases and a higher proportion of patients receiving advanced treatment lines. Liver metastases are a well-established adverse prognostic factor in gastric cancer, and real-world data indicate that only a limited proportion of patients are able to proceed to subsequent treatment lines, reflecting an inherently poorer prognosis.^2^^,^^3^ In addition, programmed death ligand-1 (PD-L1) expression is a known prognostic and predictive biomarker for immunotherapy response in metastatic gastric cancer.^4^^,^^5^ The absence of PD-L1 data further limits the interpretation of survival outcomes. Although statistical approaches such as propensity score matching could theoretically reduce baseline imbalances in retrospective analyses, incomplete data on key prognostic variables restrict the ability to adequately account for residual confounding.

The relationship between antibiotic use and immunotherapy outcomes remains complex and incompletely understood. Although results are heterogeneous, most studies have reported that antibiotic use is associated with reduced immunotherapy efficacy and shorter survival in patients with cancer; the immune-biological mechanisms are not fully understood.^6^ These complex mechanisms suggest that factors such as the type, duration, and form of antibiotics (oral or intravenous) may introduce several issues. However, these aspects are not addressed in the study, and their potential impact on the results cannot be overlooked. While the authors emphasize the study limitations, we suggest that more careful evaluations and prospective studies are needed before concluding that antibiotics improve survival in immunotherapy patients. We sincerely thank the authors for their valuable hypotheses, research, and meticulous work in this insightful study.

In conclusion, while Zhang et al.'s study provides valuable insights into the association between prophylactic antibiotic use and clinical outcomes in gastric cancer patients undergoing immunotherapy, further studies are needed to better understand the relationship between antibiotic use and immune response.

## Conflicts of interest

None declared.
